# M1 receptor modulator VU0364572 attenuates cortico-thalamo-striatal transmission and facilitates cocaine CPP extinction

**DOI:** 10.1007/s00018-026-06262-6

**Published:** 2026-06-02

**Authors:** Chao Wu, Cesar R. Romero-Leguizamón, Amir Rezagholizadeh, Morgan Thomsen, Kristi A. Kohlmeier

**Affiliations:** 1 Department of Drug Design and Pharmacology, Faculty of Health and Medical Sciences, University of Copenhagen, Universitetsparken 2, Nørre Álle 71, Copenhagen, DK-2100 Denmark; 2 Laboratory of Neuropsychiatry, Psychiatric Centre Copenhagen, Bispebjerg and Frederiksberg Hospital, Copenhagen, Denmark; 3 Department of Neuroscience, Faculty of Health and Medical Sciences, University of Copenhagen, Copenhagen, Denmark; 4 Copenhagen Center for Translational Research, Copenhagen University Hospital - Bispebjerg and Frederiksberg, Copenhagen, Denmark

**Keywords:** M1 receptor, Cortico-thalamo-striatal, Muscarinic, Acetylcholine, Cocaine use disorder

## Abstract

Cocaine use disorder (CUD), marked by persistent drug-seeking and frequent relapse, remains without an effective pharmacological therapy. Modulation of the M1 muscarinic receptor has emerged as a promising therapeutic strategy. VU0364572, a reported M1 receptor bitopic partial agonist, has been shown to reduce excitatory transmission onto the nucleus accumbens (NAc), a key region implicated in addiction. However, its effects on specific excitatory inputs to the NAc remain unclear. In this study, we combined whole-cell electrophysiology with viral circuit tracing to examine how VU0364572 modulates excitatory transmission of the prefrontal cortex (PFC)➜NAc and paraventricular nucleus of the thalamus (PVT)➜NAc pathways. We found that VU0364572 attenuates excitatory input from both PFC and PVT regions. Notably, we also observed a reduction in excitatory transmission from PFC to PVT neurons that project to the NAc, suggesting suppression of excitation within the polysynaptic PFC➜PVT➜NAc pathway. In cocaine-conditioned place preference experiments, repeated VU0364572 facilitated the extinction of cocaine-seeking behavior, suggesting an inhibitory effect on CUD behavior. Reductions in excitatory synaptic transmission in the NAc were present two weeks post administration, indicating a sustained action of VU0364572. These findings suggest that M1 receptor modulation by VU0364572 suppresses excitatory drive to the NAc through both direct (PFC➜NAc) and polysynaptic (PFC➜PVT➜NAc) pathways. Further, inhibitory actions in the NAc are persistent, which could underlie long-term behavioral effects. Taken together, our findings support the potential of VU0364572 as a therapeutic targeting both motivational and affective-related circuits in CUD.

**Graphical Abstract:**

**Figure:**
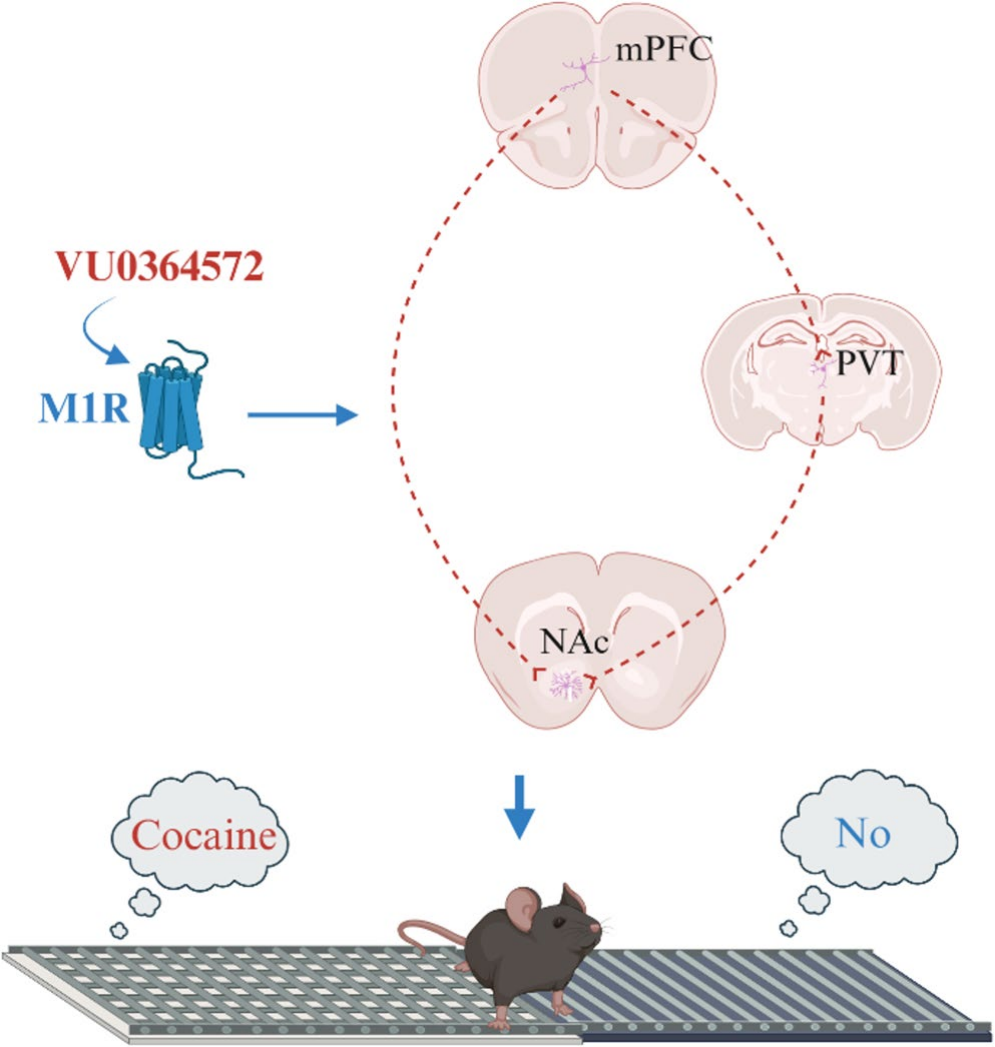
VU0364572, a reported muscarinic M1 receptor bitopic partial agonist, suppresses excitatory synaptic input through both direct (PFC➜NAc) and polysynaptic (PFC➜PVT➜NAc) pathways. Modulation of these pathways likely plays a role in VU0364572-associated facilitation of the extinction of cocaine CPP. The figure was created using BioRender. https://BioRender.com/bqenq19).

## Introduction

Cocaine is used by an estimated 25 million individuals aged 15–64 worldwide, and use prevalence has continued to increase over the past decade, accompanied by a rising mortality rate associated with cocaine use disorder (CUD) [1]. CUD is characterized by compulsive drug-seeking and a high rate of relapse [2]. Remaining abstinent when cocaine use has transitioned into CUD is a critical therapeutic challenge [3].

The abstinent state engages multiple neural systems involved in executive control, decision-making, emotional regulation, and arousal [4, 5]. Therapeutic interventions that target both motivational drive for cocaine use and the affective dysregulation associated with withdrawal may provide a promising therapeutic strategy [6, 7]. However, despite decades of research, no approved pharmacological treatments for CUD are currently available, highlighting the need for novel treatment strategies.

One promising approach involves targeting M1 muscarinic receptors, as the development of highly selective M1 modulators has revealed potential for regulating addictive behaviors. VU0364572, a reported bitopic partial agonist of the M1 muscarinic receptor [8–10], has shown promising efficacy in preclinical models. In CUD-related behaviors in rodents, VU0364572 significantly reduces cocaine self-administration and drug-seeking behavior for at least four weeks post-treatment. The behavior is accompanied by a sustained reduction in cocaine-evoked glutamate release in the nucleus accumbens (NAc) [11]. However, the cellular and synaptic mechanisms underlying VU0364572’s effects within circuits known to play a role in CUD remain largely unknown.

The NAc serves as a major hub within neural circuits implicated in the development and maintenance of CUD. The NAc receives dense excitatory inputs from multiple regions, including the prefrontal cortex (PFC), and the paraventricular nucleus of the thalamus (PVT) [12, 13]. The PFC➜NAc and PVT➜NAc projections play a critical role in regulating relapse, cue reactivity, and drug-associated memory. The PFC➜NAc pathway is primarily associated with top-down executive control, supporting goal-directed behavior and suppressing compulsive drug-seeking [14–16]. Optogenetic inhibition of prelimbic PFC input to the NAc core has been shown to effectively prevent reinstatement of cocaine-seeking behavior [17]. In contrast, the PVT conveys more emotionally salient and reward/aversion-related signals from limbic structures such as the hypothalamus and amygdala, mediating affect-driven motivation and has been implicated in the regulation of heroin and opiate addiction [18–22]. Selectively silencing this pathway reduces cocaine self-administration and cocaine-conditioned place preference (CPP) in rats [23, 24], highlighting its role in CUD regulation.

Given their potential involvement in regulating cocaine-related behaviors, M1 muscarinic receptors have attracted attention as key modulators within brain regions implicated in CUD. Anatomically, M1 receptors are abundantly expressed throughout the brain and are predominantly localized postsynaptically [25, 26]. In the NAc, M1 receptors represent the most prevalent muscarinic receptor subtype, being expressed in the majority of medial spiny neurons (MSNs) (~ 85%) and cholinergic interneurons (~ 80%). Notably, although M1 receptors are primarily postsynaptic, a small proportion are also found on axon terminals [27–29]. In the PFC, they are mainly expressed on pyramidal neurons, particularly within dendritic spines [28, 30]. While M1 receptors are present in PVT and were shown to modulate excitatory transmission [31], very little is known about their functions in PVT.

Given that the M1 receptor modulator VU0364572 inhibits cocaine-seeking behavior and M1 receptors are highly expressed in CUD-relevant excitatory circuits, we examined the effects of VU0364572 on excitatory transmission from the PFC and PVT to the NAc using whole-cell electrophysiology and viral circuit tracing. Our findings reveal that VU0364572 attenuates both PFC➜NAc and PVT➜NAc excitatory transmission, and further, our results revealed a decrease in excitatory activity within the polysynaptic PFC➜PVT➜NAc pathway. Behaviorally, VU0364572 facilitated the extinction of cocaine CPP, and effects on reducing excitatory synaptic drive to the NAc persisted for at least two weeks. These results suggest that M1 receptor modulation via VU0364572 dampens excitatory drive to NAc both directly modulated by PFC and indirectly through a polysynaptic PFC➜PVT➜NAc pathway, and that effects to dampen NAc excitatory transmissions are persistent, implicating its potential to target both motivational and affective circuits in the treatment of cocaine addiction.

## Materials and methods

### Animals

Male C57BL/6JRj mice (6–12 weeks old) were used in all experiments. Animals were either purchased directly from Janvier Laboratories (Le Genest Saint Isle, France) or bred in-house at the University of Copenhagen from Janvier-derived breeders. All procedures complied with guidelines from the Danish Ministry of Justice’s Animal Welfare Committee and adhered to Danish and European regulations on animal research and all procedures were conducted under a valid permit (for intraperitoneal (i.p.) injections: 2021-15-0201-00980; for the behavioral procedures: 2021-15-0201-00980).

### Compounds

VU0364572 was synthesized at and purchased from the Molecular Design and Synthesis Center at the Vanderbilt Institute of Chemical Biology [8, 9]. For electrophysiology, VU0364572 was bath applied at 90 µM, a concentration consistent with ex vivo studies showing VU0364572 decreases glutamatergic transmission and in vivo studies showing that VU0364572 decreases cocaine-enhanced glutamate release in NAc [11, 32]. For behavioral experiments, VU0364572 was dissolved in sterile water and administered (i.p.) at a dose of 3.2 mg/kg. Solutions were prepared fresh daily. Cocaine was obtained from Copenhagen University Hospital and used at 5 µM for bath application or dissolved in sterile 0.9% saline and administered (i.p.) at 2, 4, 8, or 16 mg/kg for behavioral experiments.

### Surgeries

The viral vectors used were, at titers 1–2 10^13^ vg/mL: pAAV-Ef1a-DIO-hChR2(E123T/T159C)-EYFP was a gift from Karl Deisseroth (viral prep #35509-AAV9, Addgene, USA), pAAV-hSyn-Cre-P2A-dTomato was a gift from Rylan Larsen (viral prep #107738 AAVrg, Addgene, USA), and pAAV-hSyn-hChR2(H134R)-EYFP was a gift from Karl Deisseroth (viral prep #26973-AAV9, Addgene, USA). Stereotaxic injections were performed under anesthesia with sevoflurane vapor (Baxter, USA) and following local lidocaine administration. Virus was injected into the PFC (500nl; AP: +1.7 mm, ML: +0.3 mm, DV: −2.25 mm), NAc (500 nl-600 nl; AP: +1.2 mm, ML: +1.25 mm, DV: −4.5 mm), and PVT (500nl; bregma − 1.56 mm, lateral + 1.10 mm, ventral − 2.90 mm at a 10-degree angle) using a stereotaxic apparatus (Kopf 1900, David Kopf Instruments, USA). Viral infusions were delivered using a 10 µl NanoFil syringe with a 33-gauge needle and microinjection pump (SMARTouch™, World Precision Instruments, USA) at a rate of 100 nl/min. The needle was left in place for 5 min following injection to minimize backflow. The incision was closed and mice were returned to their home cages. Mice received carprofen (5 mg/kg) preoperatively and for 2 days postoperatively. Viral expression was allowed for 2–4 weeks before histological verification and electrophysiology experiments.

### Slice preparation and electrophysiology

Mice were briefly anesthetized with isoflurane until unresponsive to paw pinch and then decapitated. The brains were rapidly extracted, trimmed at the brainstem, and mounted upright for slicing. Coronal brain slices (250 μm) containing NAc or PFC, or PVT were prepared using a vibratome (VT1200S, Leica, Germany) in ice-cold NMDG-based cutting solution containing (in mM): 93 NMDG, 30 NaHCO₃, 25 glucose, 20 HEPES, 10 MgSO₄, 5 Na ascorbate, 3 Na pyruvate, 2.5 KCl, 1.2 NaH₂PO₄, 0.5 CaCl₂; saturated with 95% O₂/5% CO₂). For electrical stimulation of the PFC➜NAc pathway, slices were cut at a ~ 10° angle to preserve both regions within the same slice, as previously described [33]. Slices were then incubated in oxygenated artificial cerebrospinal fluid (ACSF; in mM: 125 NaCl, 2.5 KCl, 1.3 Na₂HPO₄, 1.3 Na-ascorbate, 0.6 Na-pyruvate, 2 CaCl₂, 2 MgSO₄, 10 glucose, 25 NaHCO₃) at 32 °C for 5 min, followed by recovery at room temperature for at least 1 h before recording. Whole-cell recordings were performed in oxygenated ACSF perfused at a rate of 1 mL/min. Recording electrodes (3–8 MΩ) were fabricated using a horizontal puller (P-97, Sutter Instrument, USA) and filled with an internal solution containing (in mM): 144 K-gluconate, 2 KCl, 10 HEPES, 5 Mg-ATP, 0.2 EGTA, and 0.3 Na-GTP (pH adjusted to 7.3 with KOH).

Recordings were conducted using an EPC9 amplifier (HEKA, Germany) and processed via a Digidata 1440 A interface (Molecular Devices, USA), with data acquisition performed using AxoScope 10.2 and PatchMaster software. Signals were sampled at 10 kHz and analyzed offline using Clampfit (Molecular Devices, USA) and MiniAnalysis (Synaptosoft, USA). Neurons were visualized under an upright microscope (Olympus BX50WI, Olympus, Japan) equipped with a 12-bit, fluorescence CCD camera (Sensicam, PCO Instruments, Germany) and controlled via TILL-VISION software (TILL Photonics, Germany). Neurons with stable resting membrane potentials, holding currents ≤ 100 pA, and ≤ 20% change in access resistance were included, and recordings with fewer than 20 sEPSCs in a 30-second window were excluded. Neurons recorded in the NAc were located in the core region, and projection-specific recordings did not distinguish between core and shell regions. Following viral injection, projection-specific neurons in the NAc were identified by EYFP fluorescence and consistent, time-locked responses to 470 nm blue light stimulation (10 pulses, 5 Hz, 2 ms; Shanghai Laser & Optics Century Co., China), which was delivered to the tissue through an optical fiber (Neurophotometrics, USA ) connected via a 200 μm, 0.37 NA patch cord (Doric, Canada) to a Master-9 Pulse stimulator (A.M.P.I., Isreal). PVT neurons receiving input from the PFC and projecting to the NAc were identified by tdTomato fluorescence surrounded by EYFP labeling, together with optically evoked synaptic responses. Recordings were performed in the medial-posterior PVT, due to the role of which in cue-induced reward processing [34, 35]. Fluorescence imaging was performed using a Polychrome V monochromator (TILL Photonics, Germany) equipped with filter sets to view tdTomato (excitation 590/20 nm, emission 617/50 nm) and EYFP (excitation 340/380 nm, emission 510 nm).

The spontaneous excitatory postsynaptic currents (sEPSCs) were recorded in voltage-clamp mode at a holding potential of -60 or -70 mV. Rheobase was determined by applying 800 ms current steps in 5 pA increments to identify the minimum current required to elicit an action potential. Action potential firing was assessed using 500 ms current injections ranging from 0 to 150 pA in 10 pA steps. Paired-pulse ratio (PPR) was assessed by delivering either two 2 ms optical stimuli near the recording site or two 0.3 ms electrical stimuli in the PFC, with a 50 ms interstimulus interval and repeated 15 times. The PPR was calculated as the ratio of the second to the first evoked EPSC amplitude.

### Conditioned Place Preference (CPP) and extinction

CPP was conducted in a two-chamber apparatus with distinct visual and tactile cues using an unbiased design [36]. On day 0 (pretest), mice freely explored both chambers for 20 min to assess baseline preference. During 8 days of conditioning, mice received i.p. injections of cocaine (2, 4, 8, 16 mg/kg, increasing across sessions) or saline on alternating days and were then confined to one chamber for 30 min. Cocaine pairing was counterbalanced across chambers. During the CPP expression test and extinction tests, preference was tested following a 30 min pretreatment with an i.p. injection of VU0364572 (3.2 mg/kg) or vehicle (sterile water). We selected a 3.2 mg/kg dose of VU0364572 for CPP studies in male mice based on our previous studies on modulation of cocaine-related behaviors, in which VU0364572 was tested alone and in combination with M_4_ receptor modulators [11, 36 and unpublished data]. Mice were then allowed free exploration between the chambers for 20 min. Following the expression test, mice were returned to their home cage for 9 days to allow assessment of any delayed effects of VU0364572. A control group receiving saline paired with both chambers and pretreated with vehicle was tested alongside the test groups. CPP scores were quantified as the time spent in the cocaine-paired chamber. Acquisition was assessed by comparing CPP scores from the first post-conditioning test with pretest values, whereas extinction was assessed by comparing the CPP scores from the extinction sessions with scores from the first post-conditioning test.

### Histology and immunofluorescence

After 3–4 weeks of viral expression, mice were anesthetized with sevoflurane and transcardially perfused with 1× Phosphate-Buffered Saline (PBS) for 5 min followed by 4% paraformaldehyde (PFA) for an additional 5 min. Brains were post-fixed in PFA and then transferred to a 30% sucrose solution for cryoprotection until they sank. The tissue was embedded in OCT compound (Tissue-Tek^®^, Sakura Finetek), frozen, and sectioned at 40 μm thickness using a cryostat (CM3050 S, Leica, Germany). Sections were collected in 1× PBS, mounted onto glass slides, and coverslipped with a mounting medium (Eukitt^®^, Sigma-Aldrich, USA) to minimize fluorescence bleaching. Fluorescence images were acquired using a fluorescence microscope (Axioskop 2, Zeiss, Germany) from the PFC, NAc, or PVT, depending on the viral injection site.

### Data analysis

Electrophysiological data were analyzed using Clampfit and MiniAnalysis. sEPSC frequency and amplitude were quantified from epochs of the recording with a duration of 30 s before and after drug application. Peaks were manually verified. PPR and firing data were analyzed with Clampfit. Statistical analyses were performed using GraphPad Prism 10. Normality was assessed with the Shapiro-Wilk test. Depending on data distribution, paired or unpaired t-tests, two-way ANOVA were applied for normally distributed data, while nonparametric tests (Mann-Whitney U test, Wilcoxon matched-pairs test) were used otherwise. Repeated-measures outcomes in ANOVA were Geisser-Greenhouse corrected for departure from sphericity. For electrophysiological recordings, ‘n’ refers to the number of cells, with data obtained from at least 3 mice per experimental group to ensure reproducibility. For behavioral experiments, ‘n’ refers to the number of animals. Significance was set at *p* < 0.05.

## Results

### VU0364572 reduces baseline and cocaine-enhanced excitatory synaptic transmission in NAc

**Fig. 1:**
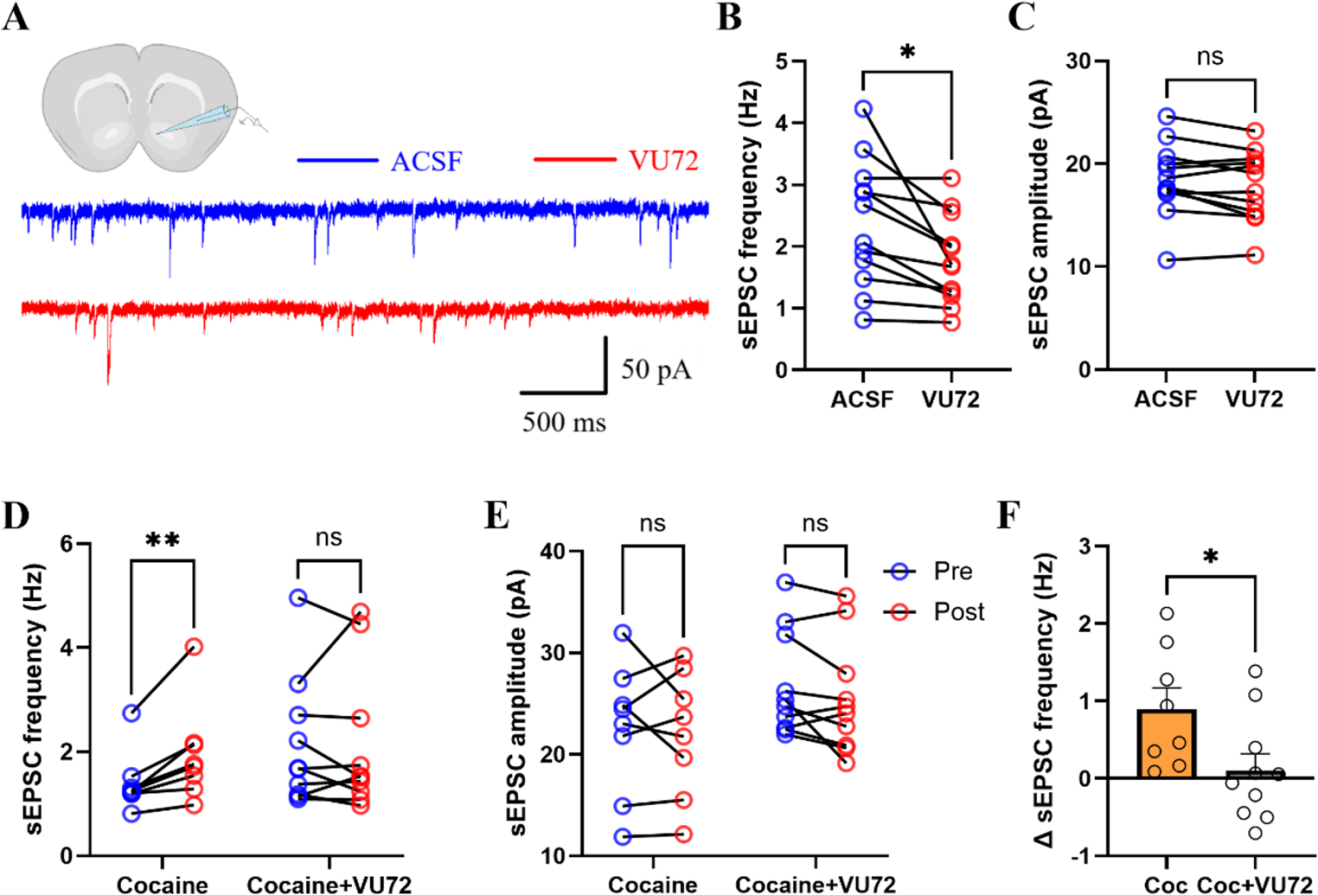
VU0364572 suppresses excitatory synaptic transmission and attenuates cocaine-induced synaptic potentiation in NAc MSNs. **A** Schematic of NAc recording site and representative traces from an MSN before and after 90 μM VU0364572 application, showing reduced frequency of sEPSCs without a change in amplitude. **B**, **C** Acute application of 90 μM VU0364572 significantly reduced the sEPSCs frequency in MSNs (**B**: paired t-test, t (11) = 3.013, *p* = 0.012) and did not significantly affect sEPSCs amplitude (**C**: paired t-test, t (11) = 1.897, *p* = 0.084; n = 12 cells from 3 mice). **D** sEPSCs frequency increased significantly after cocaine application alone (Wilcoxon matched-pairs test, W = 36, *p* = 0.008; n = 8 cells from 5 mice; blue circles indicate before treatment and red circles indicate after treatment with cocaine), but not in the cocaine + VU0364572 group (W = –11, *p* = 0.625; *n* = 10 cells from 7 mice; blue circles indicate before treatment and red circles indicate after treatment with cocaine + VU0364572). There was no statistically significant difference between the pre-cocaine baseline frequencies of the group treated with cocaine or the group treated with cocaine + VU0364572 groups (*p* = 0.1744). **E** sEPSC amplitudes were not significantly changed by either treatment (cocaine: paired t test, t(7) = 0.387, *p* = 0.710; cocaine + VU0364572: t(9) = 1.824, *p* = 0.105). The same color coding as in (**D**) is used in (**E**). **F** The cocaine-induced increase in sEPSCs frequency was significantly lower in the MSNs exposed to VU0364572 (paired t test, t (11) = 3.013, *p* = 0.012). Data are presented as individual cells and mean ± SEM. *p* < 0.05 is considered statistically significant. *p<0.05, **p<0.001, ***p<0.0001

VU0364572 has been shown to reduce cocaine-enhanced glutamate release in the NAc four weeks after i.p. administration in a microdialysis study [11], suggesting its suppressive effect on excitatory synaptic transmission directed to the NAc. Our previous work showed that acute application of 90 µM VU0364572 reduced excitatory drive onto MSNs in the male NAc in a concentration- and sex-dependent manner [32]. We wished to identify specific afferent pathways to the NAc that could be affected; therefore, while acknowledging the concentration- and sex-dependent nature of this effect, we limited the present study to male animals and a single concentration (90 µM) which we showed in previous work reduced EPSPs directed to unidentified male MSNs, to constrain the extensive viral tracing and pathway-specific analyses to a well-defined experimental condition. In the current study, before investigating specific pathways to the NAc that could be affected, we wished to replicate this effect. Accordingly, we recorded sEPSCs in male MSNs with 90 µM VU0364572 treatment. In support of our prior report, VU0364572 significantly reduced sEPSCs frequency (Fig. 1A, B; paired t-test, t (11) = 3.013, *p* = 0.012; *n* = 12 cells from 3 mice) without affecting amplitude (Fig. 1C; paired t-test, t (11) = 1.897, *p* = 0.084; *n* = 12 cells from 3 mice), confirming its suppressive modulation of excitatory inputs in the NAc.

We next examined whether VU0364572 acutely modulates cocaine-induced enhancement of excitatory transmission, given prior evidence that repeated in vivo cocaine exposure strengthens excitatory synaptic transmission in the NAc [37, 38]. Brain slices were treated with either cocaine alone or a combination of cocaine and VU0364572, and sEPSCs were recorded before and after application. In the cocaine alone group, cocaine significantly increased sEPSC frequency (Wilcoxon matched-pairs test, W = 36, *p* = 0.008; *n* = 8 cells from 5 mice), whereas this effect was absent in the cocaine + VU0364572 group (W = − 11, *p* = 0.625; *n* = 10 cells from 7 mice; Fig. 1D). No significant changes were observed in sEPSC amplitude in either group (Fig. 1E; cocaine: paired t test, t(7) = 0.387, *p* = 0.710; cocaine + VU0364572: paired t test, t(9) = 1.824, *p* = 0.105). Notably, the increase in frequency from pre- to post-treatment was significantly attenuated by VU0364572 compared to cocaine alone (Fig. 1F; Paired t test, t(11) = 3.013, *p* = 0.012), suggesting that VU0364572 reduces cocaine-induced enhancement of synaptic events in the NAc.

After confirming that VU0364572 reduces excitatory transmission directed to NAc neurons and that effects were present in the context of increased excitatory transmission induced by cocaine, we next sought to determine whether specific known excitatory afferents to the NAc were affected.

### VU0364572 decreases PFC➜NAc excitatory transmission

The PFC provides a major excitatory input to the NAc and is critically involved in top-down control and cocaine-seeking behavior [17, 39]. Thus, in order to elucidate what inputs to the NAc might be affected by VU0364572, we first investigated whether VU0364572 modulates excitatory transmission from the PFC to the NAc.

**Fig. 2:**
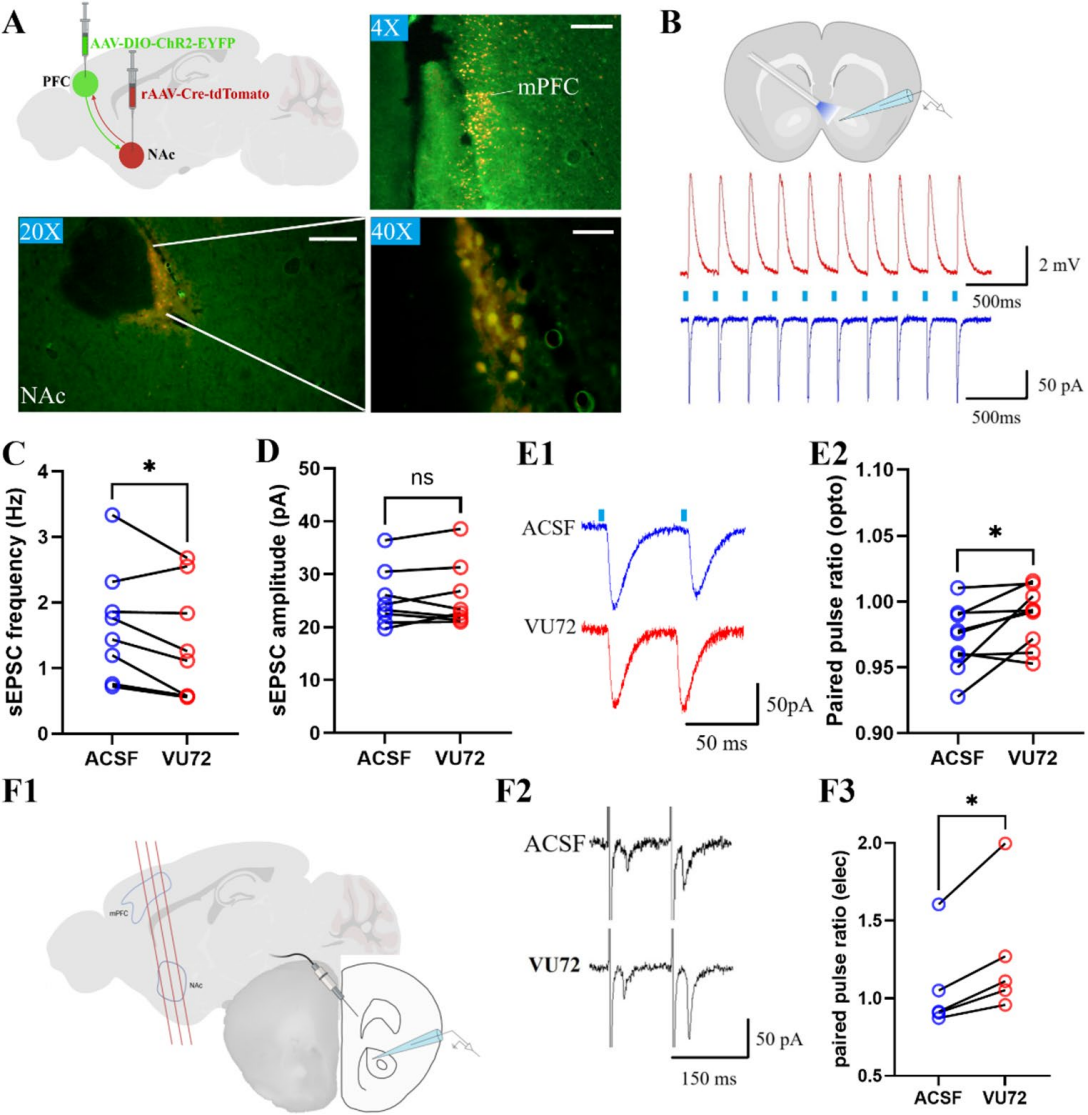
VU0364572 reduces excitatory transmission of PFC➜NAc pathway. **A** Schematic of the viral strategy for selective expression of ChR2-EYFP in the PFC➜NAc projection. A Cre-dependent ChR2-EYFP virus (AAV-DIO-ChR2-EYFP) was injected into the PFC, and a retrograde Cre-expressing virus (rAAV-Cre-tdTomato) was injected into the NAc to drive ChR2 expression specifically in PFC➜NAc projection neurons. Representative immunofluorescence image showing EYFP and tdTomato expression in the PFC, along with EYFP-labeled PFC projections and tdTomato-expressing neurons in the NAc. The tdTomato-positive neurons (40×) are encircled by EYFP-positive axons, indicating that it receives input from PFC neurons. Scale bars are 300 μm (4×), 60 μm (20×), and 30 μm (40×). **B** Schematic of the whole-cell patch-clamp recordings and optogenetic stimulation of PFC terminals in the NAc neurons. Representative traces show oEPSPs and oEPSCs following 470 nm light stimulation, confirming functional connectivity. **C**, **D** 90 μM VU0364572 reduced sEPSC frequency in NAc neurons receiving PFC input (**C**: paired t-test, t(7) = 2.564, *p* = 0.037; *n* = 8 cells from 5 mice) without affecting the amplitude (**D**: paired t-test, t(7) = 0.589, *p* = 0.575). **E** Representative traces (E1) and quantification (E2) of the paired pulse ratio (PPR) of oEPSCs before and after VU0364572 treatment. VU0364572 significantly increased PPR (t(8) = 2.497, *p* = 0.037; *n* = 9 cells from 5 mice), suggesting a reduction in presynaptic release from excitatory terminals sourcing from the PFC. **F** Schematic of the brain slice configuration illustrating electrical stimulation in the PFC and whole-cell recording in the NAc (F1). Representative traces (F2) and quantification (F3) of electrically evoked excitatory events in order to evaluate the PPR. VU0364572 significantly increased the PPR of electrically evoked EPSCs (paired t-test, t(4) = 4.007, *p* = 0.016; *n* = 5 cells from 5 mice). Data are shown as individual cells and mean ± SEM

To selectively target the PFC➜NAc projection, we employed an approach of dual virus tracing combined with optogenetics. We injected a Cre-dependent (Channelrhodopsin-2) ChR2 virus (AAV-DIO-ChR2-EYFP) into the PFC, and a retrograde Cre-expressing virus (rAAV-Cre-tdTomato) into the NAc, enabling ChR2 expression specifically in PFC➜NAc projection neurons (Fig. 2A). Immunofluorescence confirmed successful targeting, and 470 nm light reliably evoked time-locked, amplitude-consistent optogenetic excitatory postsynaptic potentials (oEPSPs) and currents (oEPSCs) across repeated stimulations in current-clamp and voltage-clamp recordings, respectively (Fig. 2A, B).

After confirming that excitatory responses could be reliably evoked, we evaluated the effects of VU0364572 on PFC➜NAc transmission by electrophysiology. We found VU0364572 significantly reduced the frequency of sEPSCs (Fig. 2C; paired t-test, t(7) = 2.564, *p* = 0.037; *n* = 8 cells from 5 mice), without affecting sEPSC amplitude (Fig. 2D; paired t-test, t(7) = 0.589, *p* = 0.575), indicating a VU0364572-mediated presynaptic reduction in excitatory neurotransmitter release from PFC terminals.

To confirm a presynaptic action of VU0364572, we evaluated the PPR using both optical and electrical stimulation of PFC afferents. VU0364572 significantly increased the PPR under both optogenetic (Fig. 2E; paired t-test, t(8) = 2.497, *p* = 0.037; *n* = 9 cells from 5 mice) and electrical stimulation conditions (Fig. 2F; paired t-test, t(4) = 4.007, *p* = 0.016; *n* = 5 cells from 5 mice), consistent with a decreased probability of excitatory neurotransmitter release from PFC presynaptic terminal in NAc.

Together, these results demonstrate that VU0364572 reduces excitatory transmission in the PFC➜NAc pathway by decreasing presynaptic release probability.

### VU0364572 decreases PVT➜NAc excitatory transmission and reduces PVT neuronal excitability

In addition to receiving direct excitatory input from the PFC, the NAc also receives glutamatergic projections from the PVT, which is located in the midline of the thalamus and receives substantial inputs from the hypothalamus, amygdala, and hippocampus, and serves as a key integrative hub for emotionally salient and arousal-related information from the limbic region [40–42]. Previous studies showed PVT inhibition decreased CUD-related behaviors in both self-administration and CPP animal models [23, 43]. We therefore investigated whether VU0364572 modulates excitatory transmission within the PVT➜NAc projection.

**Fig. 3:**
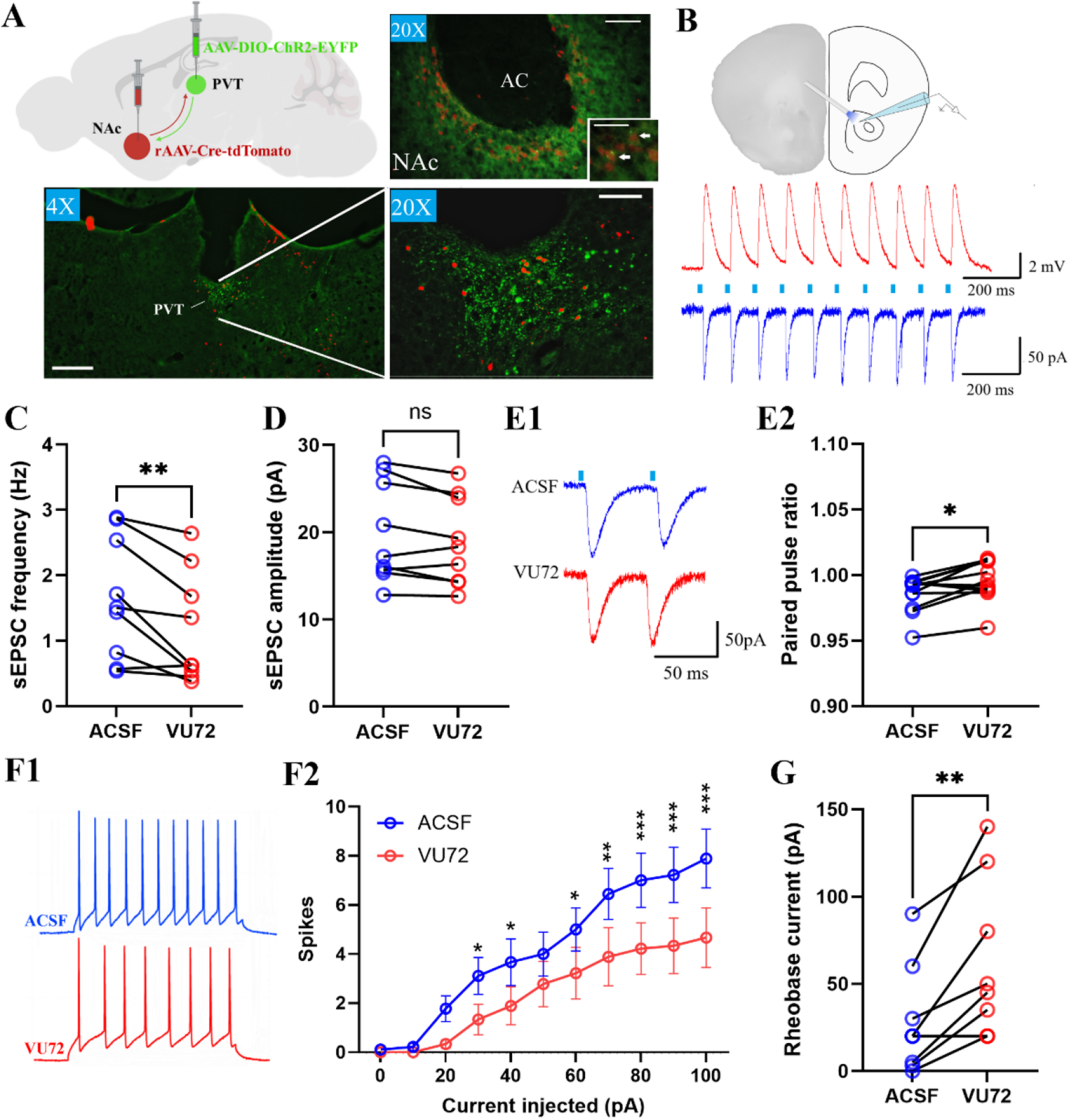
VU0364572 suppresses excitatory transmission of PVT➜NAc pathway and PVT excitability. **A** Schematic of virus injection to selectively express ChR2-EYFP in PVT➜NAc pathway. AAV-DIO-ChR2-EYFP was injected into the PVT, and rAAV-Cre-tdTomato was injected into the NAc to restrict ChR2 expression to PVT➜NAc projection neurons. Representative immunofluorescence image showing EYFP and tdTomato expression in the PVT, as well as EYFP-labeled PVT projections and tdTomato-expressing neurons in the NAc. The inset (40×) highlights tdTomato-positive neurons (arrow) surrounded by EYFP-positive axons, indicating that this neuron receives input from PVT neurons. Scale bars are 300 μm (4×), 60 μm (20×, main image), and 30 μm (insets). **B** Schematic indicating the region of whole-cell patch-clamp recordings from NAc neurons and optogenetic stimulation of PVT terminals. Representative traces show oEPSPs and oEPSCs, confirming successfully targeting of the PVT➜NAc projection neurons. **C**, **D** 90 μM VU0364572 significantly reduced the frequency of sEPSCs of PVT➜NAc projection neurons (**C**: paired t-test, t(8) = 3.562, *p* = 0.007; *n* = 8 cells from 3 mice), without significantly affecting the sEPSC amplitude (**D**: paired t-test, t(8) = 2.131, *p* = 0.066). **E** Representative traces (F1) and quantification (F2) of optogenetically evoked PPR. VU0364572 significantly increased PPR (Wilcoxon matched-pairs signed-rank test, W = 60.0, *p* = 0.016; *n* = 12 cells from 3 mice), indicating decreased presynaptic excitatory neurotransmitter release probability. **F** Representative traces showing action potential firing in PVT neurons in response to depolarizing current steps before and after VU0364572 treatment (F1). VU0364572 significantly reduced spike firing in PVT neurons (two-way ANOVA, main effect of treatment: F(1,8) = 9.640, *p* = 0.015; *n* = 9 cells from 6 mice) (F2). **G** Rheobase current was significantly increased following VU0364572 application (paired t-test, t(8) = 3.589, *p* = 0.007; *n* = 9 cells from 6 mice), indicating reduced intrinsic excitability of PVT neurons. Data are presented as individual cells and mean ± SEM

To selectively label this circuit, we injected AAV-DIO-ChR2-EYFP into the PVT and rAAV-Cre-tdTomato into the NAc to enable ChR2 expression specifically in PVT➜NAc projection neurons (Fig. 3A). Immunofluorescence imaging along with time-locked and amplitude-consistent oEPSPs and oEPSCs, confirmed successful targeting and functional activation of the pathway (Fig. 3A and B).

Whole-cell recordings from PVT➜NAc projection neurons revealed that VU0364572 also significantly reduced the frequency of sEPSCs (Fig. 3C; paired t-test, t(8) = 3.562, *p* = 0.007; *n* = 8 cells from 3 mice), without affecting amplitude (Fig. 3D; paired t-test, t(8) = 2.131, *p* = 0.066; *n* = 8 cells from 3 mice), suggesting a presynaptic reduction in excitatory neurotransmitter release from PVT. Consistent with this interpretation, PPR analysis showed a significant increase in the ratio of the first and 2nd oEPSCs following VU0364572 application (Fig. 3E, Wilcoxon matched-pairs signed-rank test, W = 60.0, *p* = 0.016; *n* = 12 cells from 3 mice), indicating a decreased probability of excitatory neurotransmitter release from PVT terminals in the NAc.

We next asked whether these changes were associated with altered PVT neuronal activity, so we then recorded action potential firing in PVT neurons in response to depolarizing current steps to detect the activity changes of PVT neurons. We found VU0364572 significantly reduced spike firing in PVT neurons (Fig. 3F; two-way ANOVA, main effect of VU0364572: F(1,8) = 9.640, *p* = 0.015), with Tukey’s post hoc analysis revealing reductions at 30, 40, 60, 70, 80, 90, and 100 pA (30 pA: *p* = 0.025; 40 pA: *p* = 0.025; 60 pA: *p* = 0.025; 70 pA: *p* = 0.002; 80 pA: *p* = 0.0006; 90 pA: *p* = 0.0004; 100 pA: *p* < 0.0001; *n* = 9 cells from 6 mice). In addition, VU0364572 increased rheobase current (Fig. 3G; paired t-test, t(8) = 3.589, *p* = 0.007; *n* = 9 cells from 6 mice), consistent with a reduction in the excitability of PVT neurons.

Taken together, these findings demonstrate that VU0364572 reduces PVT➜NAc excitatory transmission by decreasing presynaptic release and lowering the intrinsic excitability of PVT neurons. Combined with its effects on the PFC➜NAc pathway, these results suggest that VU0364572 attenuates both excitatory drive from PFC and PVT to the NAc.

### VU0364572 reduces activity in the PFC➜PVT➜NAc circuit and reduces PFC neuronal excitability

**Fig. 4:**
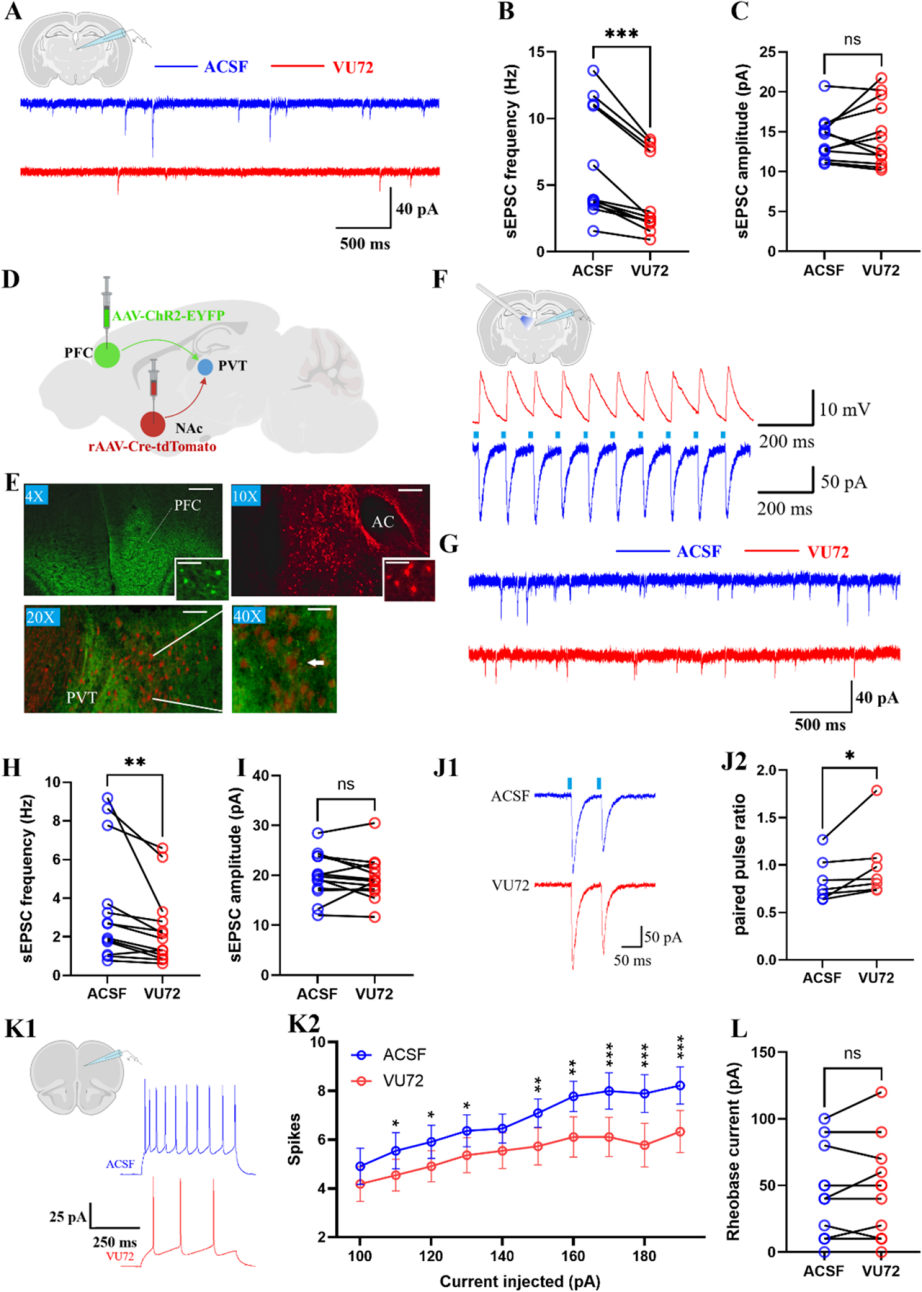
VU0364572 suppresses activity in the PFC➜PVT➜NAc circuit and reduces PFC neuronal excitability. **A** Representative recording from a PVT neuron showing sEPSCs before and after application of 90 µM VU0364572. VU0364572 application reduces sEPSC frequency without affecting amplitude. **B** Quantification showing that VU0364572 significantly decreased sEPSC frequency in PVT neurons (paired t test, t(11) = 3.589, *p* < 0.001; *n* = 12 cells from 6 mice). **C** sEPSC amplitude remained unchanged following VU0364572 treatment (paired t test, t(11) = 0.882, *p* = 0.397). **D** Schematic and representative images showing the dual-labeling strategy: AAV-ChR2-EYFP was injected into the PFC, and retrograde rAAV-Cre-tdTomato into the NAc, allowing identification of PVT neurons that receive excitatory input from the PFC and project to the NAc. **E** Immunofluorescence images showing EYFP expression in the PFC and tdTomato expression in the NAc. In the PVT, EYFP-labeled projections from the PFC can be seen in close apposition to tdTomato-expressing neurons that project to the NAc. The arrow in the 40× image highlights a tdTomato-positive PVT neuron surrounded by EYFP-positive axons from the PFC, indicating that this neuron receives input from the PFC and sends projections to the NAc. Scale bars are 300 μm (4×, main image), 120 μm (10×, main image), 60 μm (20×), and 30 μm (40× and insets). **F** Schematic of whole-cell patch-clamp recordings from PVT neurons and optogenetic stimulation of PFC terminals. These PVT➜NAc neurons receiving PFC projection exhibited time-locked, amplitude-consistent oEPSPs and oEPSCs in response to PFC stimulation. **G** Example of a sEPSC trace before and after 90 µM VU0364572 from a PVT➜NAc neuron receiving PFC input. VU0364572 reduced the frequency of sEPSCs without altering the amplitude. **H** In these identified neurons, VU0364572 significantly reduced sEPSC frequency (Wilcoxon matched-pairs test, W = -85, *p* = 0.0012; *n* = 13 cells from 7 mice). **I** sEPSC amplitude was not significantly affected (paired t-test, t(12) = 0.570, *p* = 0.579; *n* = 13 cells from 7 mice). **J** Representative traces (J1) and quantification (J2) of optogenetically evoked PPR. Analysis showed an increased second-to-first oEPSC ratio after VU0364572 treatment (Wilcoxon matched-pairs signed-rank test, W = 28, *p* = 0.016; *n* = 7 cells from 4 mice), suggesting reduced presynaptic excitatory neurotransmitter release probability at PFC terminals. **K** Representative trace from a PFC neuron showing that VU0364572 induced a reduction in action potential firing (K1). Whole-cell recordings demonstrated that VU0364572 significantly decreased firing rates (F(1,10) = 9.270, *p* = 0.012; *n* = 11 cells from 4 mice) (K2). **L** Rheobase current was unaffected (paired t-test, t(10) = 0.896, *p* = 0.391; *n* = 11 cells from 4 mice), indicating that VU0364572 suppresses PFC neuronal excitability without altering the threshold for action potential initiation. Data are presented as individual cells and mean ± SEM

We wished to further explore the mechanism underlying VU0364572 induced suppression of PVT activity. Given that both the PFC➜NAc and PVT➜NAc pathways showed presynaptic reductions, we tested whether the suppression of PVT activity was associated with decreased excitatory input onto PVT neurons. Whole-cell recordings from PVT neurons revealed that VU0364572 significantly decreased the frequency of sEPSCs (Fig. 4B; paired t-test, t(11) = 3.589, *p* < 0.001), without affecting their amplitude (Fig. 4C; paired t-test, t(11) = 0.882, *p* = 0.397; *n* = 12 cells from 6 mice), indicating a reduction of activity of PVT neurons arose from decreased excitatory synaptic input directed onto PVT neurons in presence of VU0364572.

One of the major sources of excitatory input to the PVT arises from glutamate neurons in the PFC, which play a critical role in cue-reward learning [34, 44]. As we already showed that VU0364572 decreased excitatory transmission in both PFC➜NAc and PVT➜NAc, we therefore hypothesized that the PVT➜NAc decrease may arise from the effect from PFC neurons, and form a polysynaptic PFC➜PVT➜NAc pathway regulation. To test this, we injected a non Cre-dependent AAV-ChR2-EYFP into the PFC and rAAV-Cre-tdTomato into the NAc, and recorded in PVT (Fig. 4D&E), by which we could assess the changes of PVT neurons that receive afferents from PFC and project to NAc. Those neurons were identified by being both tdTomato-positive and exhibiting time-locked, amplitude-consistent oEPSPs and oEPSCs (Fig. 4F).

In these dual-labeled PVT neurons, VU0364572 significantly reduced the frequency of sEPSCs (Fig. 4H; Wilcoxon matched-pairs test, W = -85, *p* = 0.0012; *n* = 13 cells from 7 mice) without altering their amplitude (Fig. 4I; paired t-test, t(12) = 0.570, *p* = 0.579; *n* = 13 cells from 7 mice), suggesting a reduced presynaptic input from the PFC. Consistently, PPR analysis revealed an increased ratio of the second to the first oEPSC (Fig. 4J, Wilcoxon matched-pairs signed-rank test, W = 28, *p* = 0.016; *n* = 7 cells from 4 mice), indicating a reduced release probability at PFC terminals to PVT➜NAc neurons, confirming a downregulation of the PFC➜PVT➜NAc pathway.

Because VU0364572 induced decreases in both PFC➜NAc and polysynaptic PFC➜PVT➜NAc pathways, we next asked if VU0364572 decreased activity in PFC excitatory neurons. We measured action potential firing in response to depolarizing current steps. We found that VU0364572 significantly inhibited spike firing (Fig. 4K; two-way ANOVA, main effect of VU0364572: F(1,10) = 9.270, *p* = 0.012; *n* = 11 cells from 4 mice), with Tukey’s post hoc tests showing reductions at 110–130 pA and 150–190 pA current injections (110 pA: *p* = 0.037; 120 pA: *p* = 0.037; 130 pA: *p* = 0.037; 150 pA: *p* = 0.005; 160 pA: *p* = 0.002; 170 pA: *p* = 0.0004; 180 pA: *p* < 0.0001; 190 pA: *p* = 0.0004). However, the rheobase current was unchanged (Fig. 4L; paired t-test, t(10) = 0.896, *p* = 0.391; *n* = 11 cells from 4 mice), indicating that VU0364572 suppresses excitability without altering the threshold for action potential initiation.

Collectively, these findings show that VU0364572 reduces excitatory drive to the NAc through both direct (PFC➜NAc) and polysynaptic (PFC➜PVT➜NAc) pathways by dampening excitatory transmission and neuronal excitability across multiple circuit nodes.

### VU0364572 accelerates cocaine CPP extinction and induces long-lasting suppression of excitatory transmission onto NAc MSNs

**Fig. 5:**
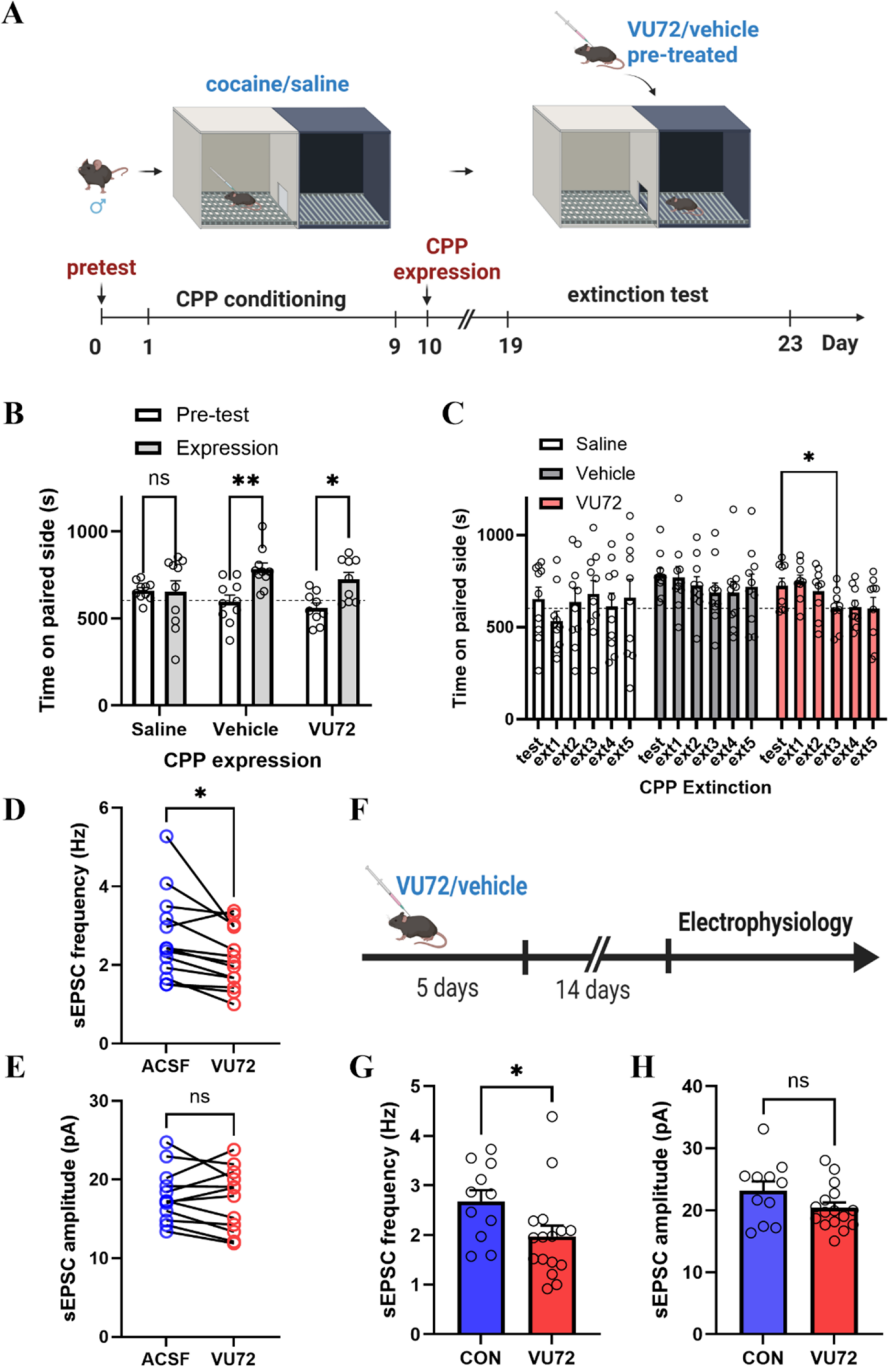
VU0364572 Facilitates Cocaine CPP Extinction and Induces Long-Term Suppression of Excitatory Transmission onto NAc MSNs. **A** Schematic of the cocaine CPP training and extinction protocol. Created in BioRender (https://BioRender.com/37kusv0). **B** No significant difference in cocaine preference between animals that later received VU0364572 and those that received vehicle in the first CPP expression test (Holm-Šidák post hoc test; saline: *p* = 0.88; vehicle: *p* = 0.008; VU0364572: *p* = 0.02; *n* = 9-10 per group). **C** VU0364572 significantly reduced the CPP score in the 3rd test of extinction, while vehicle-treated mice maintained their CPP (One way ANOVA, VU0364572:F(5,40) = 4.14, *p* = 0.02); vehicle: F(5,45) = 1.83, *p* = 0.14; Dunnett’s posthoc test; vehicle: P_ext1_ = 0.998, P_ext2_ =0.508, P_ext3_ = 0.071, P_ext4_ = 0.184, P_ext5_ = 0.661; VU0364572: P_ext1_ = 0.878, P_ext2_ = 0.884, P_ext3_ = 0.039, P_ext4_ = 0.113, P_ext5_ = 0.324; *n* = 9-10 per group). **D** Acute application of VU0364572 significantly reduced the frequency of sEPSCs in NAc MSNs from cocaine conditioned mice (paired t-test: t(12) = 2.83, *p* = 0.015; *n* = 13 cells from 4 mice). **E** When acutely applied, VU0364572 did not significantly alter the amplitude of sEPSCs in NAc MSNs from cocaine conditioned mice (paired t-test: t(11) = 0.72, *p* = 0.487; *n* = 12 cells from 4 mice). **F** Experimental timeline for electrophysiological recordings. Created in BioRender (https://BioRender.com/3x9g30w). **G** VU0364572-treated mice showed a significant reduction in the frequency of sEPSCs in NAc MSNs compared to controls two weeks after VU0364572 treatment (Mann-Whitney U = 40, *p* = 0.017; *n* = 27 cells from 7 mice). **H** No significant difference was observed between groups for the amplitude of sEPSCs (unpaired t-test, t(25) = 1.667, *p* = 0.108; *n* = 27 cells from 7 mice). Ext: Extinction. Data are presented as individual subjects (**B**, **C**) or cells (**E**, **F**) and mean ± SEM

Inhibiting PVT neurons has been shown to prevent the expression of cocaine CPP [43] and suppress activity in excitatory projections from the PFC➜NAc reduces cue-induced reinstatement of cocaine seeking [17, 45]. Optogenetic silencing the PVT➜NAc pathway induced long-lasting disruption of morphine CPP expression but not acquisition and prevented morphine-prime-induced reinstatement of morphine-conditioned CPP [46]. Further, inhibition of a pathway from the PFC to the PVT decreased cue-induced reinstatement of drug-seeking behavior in cocaine-experienced rats [47]. Taken together, attenuation of excitatory signaling in the PFC➜NAc and PVT➜NAc pathways by VU0364572 would be expected to alter neural processes driving motivated behavior, which could influence expression of cocaine CPP behavior. To test this prediction, we examined whether systemic VU0364572 would facilitate the extinction of cocaine-CPP behavior (Fig. 5A).

Mice underwent conditioning in which they received either i.p. cocaine or saline injection in different chambers. Cocaine preference was assessed by measuring the time spent in the cocaine-paired chamber versus the total time. After eight days of conditioning, both VU0364572- and vehicle-treated groups showed CPP in the first expression of CPP test, while saline controls showed no side preference (Fig. 5B; two-way ANOVA, main effect of test phase: F(1,26) = 11.92, *p* = 0.002; main treatment effect: F(2,26) = 0.71, *p* = 0.50; treatment by session interaction: F(2,26) = 3.55, *p* = 0.04; Holm-Šidák posthoc test: saline: *p* = 0.88; vehicle: *p* = 0.008; VU0364572: *p* = 0.02; *n* = 9–10 per group). However, in accordance with our prediction, during extinction testing VU0364572 exerted a significant effect on CPP (Fig. 5C; two-way ANOVA, main effect of session: F(5,130) = 2.10, *p* = 0.10; main treatment effect: F(2,26) = 1.04, *p* = 0.37; treatment by session interaction: F(10,130) = 2.54, *p* = 0.02). Specifically, VU0364572 reduced preference for the previously cocaine-paired environment (one-way ANOVA, effect of session: F(5,40) = 4.14, *p* = 0.02), compared to vehicle-treated mice, who maintained their preference for at least 5 sessions (F(5,45) = 1.83, *p* = 0.14). In parallel with these behavioral findings, acute application of VU0364572 reduced excitatory input onto NAc MSNs from mice that experienced the same 8-day cocaine conditioning, as indicated by a decrease in sEPSC frequency (Fig. 5D; paired t-test: t(12) = 2.83, *p* = 0.015; *n* = 13 cells from 4 mice), without a significant change in sEPSC amplitude (Fig. 5E; paired t-test: t(11) = 0.72, *p* = 0.487; *n* = 12 cells from 4 mice). These results indicate that VU0364572 facilitates the extinction of cocaine seeking, consistent with decreased cocaine seeking or decreased control of drug-associated cues over behavior, potentially by reducing excitatory synaptic input onto NAc MSNs.

We previously found that VU0364572 reduces cocaine self-administration weeks after exposure which is associated with a sustained reduction in cocaine-evoked glutamate release in NAc [11]. When combined with our CPP data, these findings indicate that VU0364572 exerts persistent effects on both cocaine-related behaviors and underlying neural mechanisms. However, whether VU0364572 produces lasting changes in excitatory synaptic transmission within the NAc has not been established. To address this, mice received daily i.p. injections of VU0364572 or vehicle for five consecutive days, followed by a two-week drug-free period before ex vivo, whole-cell recordings (Fig. 5F). Compared with controls, VU0364572-treated mice exhibited a significant reduction in the frequency of sEPSCs in NAc MSNs (Fig. 5G; Mann-Whitney U = 40, *p* = 0.017; *n* = 27 cells from 7 mice), with no difference in sEPSC amplitude (Fig. 5H; unpaired t-test, t(25) = 1.667, *p* = 0.108).

Together, these results demonstrate that VU0364572 facilitates the extinction of cocaine-context associations. At the synaptic level, VU0364572 produces a long-lasting suppression of excitatory input to NAc MSNs.

## Discussion

Previous studies have shown that the selective M1 muscarinic receptor modulator VU0364572 decreases glutamatergic transmission and attenuates cocaine-induced elevations of extracellular glutamate in the NAc [11, 32]. In the present study, we investigated the synaptic mechanisms underlying these effects by examining how VU0364572 modulates excitatory inputs to the NAc. We found that VU0364572 significantly suppresses excitatory synaptic transmission in both the PFC➜NAc pathway, which mediates top-down cognitive control and cue processing [17, 45, 48, 49], and the PVT➜NAc pathway, which conveys affective and context/drug association-related signals [19, 42, 50, 51]. Furthermore, we also demonstrate for the first time that VU0364572 modulates the PFC➜PVT➜NAc circuit, suggesting that M1 receptor activation can influence both direct and indirect polysynaptic cortical regulation of the NAc. Behaviorally, VU0364572 significantly accelerated cocaine CPP extinction. At the synaptic level, VU0364572 decreased excitatory transmission onto NAc MSNs in cocaine conditioned mice and five days of VU0364572 treatment in vivo produced a long-lasting suppression of excitatory transmission onto NAc MSNs that persisted for at least two weeks. Together, these findings reveal a circuit-specific role for M1 receptor modulation in dampening excitatory drive within cortico-thalamo-striatal circuits, supporting its therapeutic potential for reducing maladaptive drug-seeking behaviors in CUD.

The NAc integrates major excitatory inputs from both the PFC and the PVT, forming key components of the neural circuits that regulate drug-seeking behavior [52]. Use of retrograde tracing and electrophysiological characterization suggests that mPFC neurons projecting to the NAc and PVT constitute largely distinct and anatomically segregated populations within the dorsomedial PFC. Specifically, PFC-NAc neurons are predominantly located in layers II/III (46% layer II/III, 51% layer V, 4% layer VI), whereas PFC-PVT neurons are primarily localized to layer V/VI (0% layer II/III, 20% layer V, 79% layer VI) [44]. The finding suggests minimal collateralization between mPFC-NAc and mPFC-PVT projection neurons. This anatomical segregation implies that mPFC and PVT inputs may exert complementary but distinct influences over NAc activity.

The PFC is a critical hub for cognitive control, decision making, emotional regulation, and behavioral inhibition. It encodes reward-related cues and transmits executive and goal-directed signals to the NAc, exerting top-down control over motivated behavior [48, 49]. Functional studies have demonstrated that inhibition of the prelimbic PFC projection to the NAc core attenuates cue-induced reinstatement of cocaine seeking [17, 39, 45], highlighting its role in the encoding and maintenance of cocaine-associated cues and motivational salience. Through its projections to NAc, the PVT contributes to the persistence of drug-associated memories, thereby increasing susceptibility to relapse [46]. Additionally, the PVT’s role in processing arousal signals suggests that it may modulate processing of drug cues, providing bottom-up input that influences motivational drive, thereby further influencing drug-related behaviors [53]. Silencing the PVT➜NAc pathway reduced both cocaine CPP and self-administration in rats [23, 43], highlighting its role in drug-seeking behaviors. Our finding that VU0364572 suppresses excitatory synaptic transmission in both the PFC➜NAc and PVT➜NAc pathways suggests that M1 receptor activation can dampen the integration of both cognitive and affective information in the NAc. This dual modulation may underlie the drug’s ability to disrupt maladaptive drug-seeking behavior and support extinction learning.

We further observed that VU0364572 reduced excitatory transmission within the PFC➜PVT➜NAc pathway. Projections from the PFC influence PVT activity and thus, our data suggests that VU0364572 modulates not only the direct PFC➜NAc pathway but also exerts indirect polysynaptic inhibitory control over NAc activity via inhibition of thalamic projections to the NAc. This circuit has been proposed as a convergence hub for cognitive and affective processing, integrating executive control from the PFC with emotionally salient input from the PVT [34, 42, 51]. Activation of the PFC➜PVT➜NAc circuit has been shown to suppress cue discrimination and cue-reward [34], emphasizing its role in addiction-related behavior. Although we cannot conclude that the PFC neurons modified by VU0364572 were projecting onto the same PVT cells that innervate the NAc in all cases, our final viral tracking study showed that PFC excitatory input to PVT neurons projecting to the NAc was reduced. This suggests that PFC modulation may influence downstream NAc function through reduced PVT output.

In our study, we did not localize the relevant M1 receptors targeted by VU0364572. However, we observed decreased sEPSC frequency and increased PPR in neurons from the NAc and PVT following VU0364572 treatment, indicating a presynaptic inhibitory effect. Furthermore, reduced neuronal activity in the PVT and PFC suggests that the inhibition of excitatory transmission in the NAc may originate from decreased activity in these upstream regions, particularly the PFC, which also projects to PVT and where we also observed reduced excitatory transmission from the PFC to the PVT➜NAc neurons. These findings are consistent with a previous study showing that VU0364572 induces long-term depression and reduces afferent drive into the PFC from the hippocampus and basolateral amygdala [54], though Digby et al. [9] reported that VU0364572 did not alter the frequency of sEPSCs in the PFC.

The cellular mechanisms underlying the inhibitory effects induced by VU0364572 on presynaptic inputs remain largely unclear. Activity of M1Rs can elevate intracellular Ca²⁺ levels and stimulate postsynaptic endocannabinoid signaling, which in turn suppresses presynaptic glutamate release, suggesting that VU0364572 may exert feedback inhibition on presynaptic neurons in the PFC via cannabinoid pathways [55, 56]. In addition, Yi et al. [57] reported that muscarinic actions of M1Rs enhances the activity of parvalbumin-expressing interneurons in PFC slices, implying a potential contribution of GABAergic interneurons to this inhibitory regulation. Taken together, our findings suggest that VU0364572 dampens PFC excitability resulting in reductions in excitatory output to projections involved in CUD, including the NAc and PVT. Future studies are warranted to clarify the mechanisms underlying the reduction in excitatory transmission within the PFC and the PVT, including the potential involvement of specific presynaptic cell types such as interneurons or projection neurons.

At the behavioral level, our current finding that VU0364572 facilitates extinction of cocaine CPP extends our previous work evaluating the potential of this compound and similar M1 modulators to influence addiction-related behaviors. We previously reported that M1 stimulation facilitates extinction and prevents both acquisition of cocaine self-administration and reinstatement of cocaine seeking, weeks after the treatment, in operant assays, though in mice only when combined with muscarinic M4 receptor stimulation [36, 58]. Further, we showed that acute administration of VU0364572 reduced cocaine self-administration more strongly four weeks after administration than immediately following treatment in a cocaine-food choice paradigm in rats [11]. VU0364572 and other M1 agonists or PAMs also modified the discriminative stimulus of cocaine (its subjective perception) and suppressed cocaine self-administration acutely in mice [59]. Despite these effects, VU0364572 did not alter the acquisition of cocaine CPP in an earlier study [36], nor the initial expression of cocaine CPP in the present investigation. However, here we reveal selective M1 modulation is sufficient to alter reward-related behaviors in the CPP paradigm. We cannot exclude that testing additional doses of VU0364572 and other M_1_ receptor-acting compounds would reveal more consistent effects across self-administration and CPP behaviors, as several studies, including the present one, tested a single dose only. Taken together, these findings suggest that although VU0364572 can suppress voluntary cocaine taking immediately upon administration, consistent with its acute effects on excitatory synaptic transmission in the present study, its behavioral effects are persistent. Indeed, the previous cocaine choice findings and our CPP results suggest that some effects of VU0364572 develop over days or weeks following administration. This suggests some VU0364572 induced neuroplastic changes.

Consistent with the above hypothesis, we also observed a sustained suppression of excitatory synaptic transmission by VU0364572: two weeks after systemic administration of VU0364572, electrophysiological recordings revealed a reduction in sEPSC frequency in NAc MSNs. This finding is consistent with a previous microdialysis study showing a sustained reduction in cocaine-induced glutamate release in the NAc [11]. While speculative, these results provide a mechanistic basis for behavioral data demonstrating that VU0364572 treatment leads to prolonged reductions in cocaine self-administration and cocaine-seeking in single-reinforcer and cocaine-food choice paradigms [11, 36]. These findings suggest that VU0364572 produces long-lasting attenuation of cocaine-seeking behavior by persistently dampening NAc excitatory drive, highlighting the therapeutic potential of targeting this pathway for sustained treatment effects in addiction. Interestingly, by dampening PFC excitability, VU0364572 may not only mitigate the excessive cortical activity associated with cocaine and drug-associated cues, but also avoid the cognitive impairments and convulsant activity typically observed with excessive M1R-mediated excitation, positioning VU0364572, or other partial M1 agonists like VU0364572 as safer and more effective alternatives to other M1-acting compounds shown to enhance excitability [60–62]. However, while VU0364572 has proven valuable as a preclinical M1-acting tool compound, potential lower selectivity at human M1 receptors, potential side effects and complex dose-response relationships may limit its suitability as a clinical drug candidate. Therefore, successful clinical translation will require further medicinal chemistry optimization, requiring a better understanding of the mechanism underlying VU0364572’s effects, for which our findings offer an initial framework.

This study has several limitations. First, we did not examine sex differences or concentration-response relationships for specific excitatory transmissions. Previous studies have shown that VU0364572 produces distinct cellular effects depending on concentration and exhibits sex-dependent differences at both cellular and behavioral levels [32, 36, 63–65]. Future studies should systematically evaluate the effects of concentration and sex across different excitatory pathways. Second, our investigation focused exclusively on excitatory synaptic transmission, leaving the actions on inhibitory transmission unexplored, and we failed to identify potential cellular mechanisms leading to reductions in excitatory presynaptic input, which at this time remains speculative and awaits experimental focus. Third, in this study, our focus was on evaluation of identified presynaptic input directed to MSNs, thus we did not evaluate the actions of VU72 on postsynaptic MSNs. However, in prior work, we showed that VU72 activates a potassium channel in unidentified MSNs [32], and future studies could confirm this in circuit identified MSNs. Fourth, the long-term behavioral and synaptic consequences of longer chronic VU0364572 administration remain unknown and need to be explored. Fifth, our study focused on the NAc core and middle-posterior PVT due to the role of these regions in cue-induced reward processing [34, 35]. However, because the core and shell of the NAc and the anterior and posterior PVT exhibit distinct connectivity patterns [66], a more comprehensive investigation across the NAc and PVT subregions is warranted. Finally, although we observed VU0364572-induced suppression of excitatory transmission in key addiction-related circuits, establishing a causal relationship with behavioral outcomes will require targeted manipulations using cell- and region-specific approaches.

In conclusion, our findings reveal a circuit-selective inhibitory effect of VU0364572 on excitatory transmission within the PFC➜NAc and PFC➜PVT➜NAc axis. Reductions in EPSPs in the NAc were long-lasting. These neuromodulatory actions were accompanied by a diminished impact of cocaine on sEPSCs in the NAc and VU0364572 also facilitated the extinction of cocaine CPP. Together, our results support a novel therapeutic mechanism by which M1 receptor modulation may recalibrate cortico-striatal-thalamic circuits to promote adaptive behavioral flexibility. The ability of VU0364572 to modulate both cognitive and affective pathways highlights its promise as a candidate intervention for relapse prevention in CUD.

## Data Availability

Data is held in a repository at the University of Copenhagen and is available upon request made to the corresponding author.
